# The Potential Role of Immune Alteration in the Cancer–COVID19 Equation—A Prospective Longitudinal Study

**DOI:** 10.3390/cancers12092421

**Published:** 2020-08-26

**Authors:** Tal Goshen-Lago, Moran Szwarcwort-Cohen, Madeleine Benguigui, Ronit Almog, Ilit Turgeman, Nelly Zaltzman, Michael Halberthal, Yuval Shaked, Irit Ben-Aharon

**Affiliations:** 1 Division of Oncology, Rambam Health Care Campus, Haifa 31096, Israel; T_GOSHENLAGO@Rambam.Health.gov.il (T.G.-L.); i_turgeman@rambam.health.gov.il (I.T.); 2 Virology Laboratory, Rambam Health Care Campus, Haifa 31096, Israel; m_szwarcwort@rambam.health.gov.il (M.S.-C.); n_zaltzmanbershadsky@rambam.health.gov.il (N.Z.); 3 Rappaport Faculty of Medicine, Technion, Haifa 3525428, Israel; madeleine.benguigui@gmail.com (M.B.); r_almog@rambam.health.gov.il (R.A.); m_halberthal@rambam.health.gov.il (M.H.); yshaked@technion.ac.il (Y.S.); 4 Epidemiology Department and Biobank, Rambam Health Care Campus, Haifa 31096, Israel; 5 General Management, Rambam Health Care Campus, Haifa 31096, Israel; 6 Rappaport-Technion Integrated Cancer Center, Technion, Haifa 3525428, Israel

**Keywords:** COVID19, serology, cancer

## Abstract

**Simple Summary:**

Despite lack of concrete evidence, cancer patients were considered at the onset of the COVID19 pandemic as high-risk population for COVID19 infection. However, current evidence is inconclusive and the potential role of cancer or anti-neoplastic treatments in COVID19 course remains to be elucidated. Our results may indicate that due to differential immune cell profile of cancer patients who are treated with immunomodulatory agents the host response to the SARS-COV2 may lessen symptom severity. Delineating COVID-19 infection trends in asymptomatic healthcare workers as well as a cohort of cancer patients who are on active anti-cancer treatment will lend credence to tailor future healthcare policy in the next phases of the pandemic.

**Abstract:**

*Background:* The risk of cancer patients to develop COVID19 infection is unclear. We aimed to prospectively study cancer patients and oncology healthcare workers for COVID19 serology. In IgG+ cases, immune profile was determined to portray the pattern of immune response to SARS-CoV2. *Methods:* Cancer patients on active treatment and healthcare workers were enrolled. During the study period (3/2020–6/2020), demographic data and blood were collected at three time points. Expression of IgG, IgM, and IgA were assessed. In SARS-CoV-2 IgG+ cases and matched negative cases, we performed mass cytometry time of flight (CyTOF) analysis on the basis of the expression of surface markers. *Results:* The study included 164 cancer patients on active intravenous treatment and 107 healthcare workers at the cancer center. No symptomatic cases were reported during the study period. Serology analysis revealed four IgG+ patients (2.4%) and two IgG+ healthcare workers (1.9%)—all were asymptomatic. CyTOF analysis demonstrated substantial reduction in myeloid cells in healthcare workers who were SARS-CoV-2 IgG+ compared to those who were SARS-CoV-2 IgG-, whereas in cancer patients, the reduction was relatively milder (≈50% reduction in SARS-CoV-2 IgG+ cancer patients compared with ≈90% reduction in SARS-CoV-2 IgG+ workers). *Conclusion:* Our results indicate a similar rate of asymptomatic COVID19 infection in cancer patients and healthcare workers in a longitudinal study throughout the pandemic time. Due to differential immune cell profiles of cancer patients who are treated with immunomodulatory agents, the host response to the SARS-COV2 may play a role in COVID19 course and representation. The immunological perspective of cancer treatments on the risk for COVID19 infection should be further explored.

## 1. Introduction

The outbreak of coronavirus disease 2019 (COVID19) pandemic has introduced a substantial healthcare challenge globally. COVID19 morbidity and mortality have been correlated with old age and comorbidities, leading to poorer clinical outcomes and occasionally resulting in hospitalization and higher likelihood for intubation [1]. Hypertension had been found as the leading risk factor predispose for COVID19 infection [2], while other supposed potential risk factors at the early phase of the pandemic had been either refuted or partially confirmed. Cancer patients were at first generally postulated as a large subgroup at high risk of developing COVID19 infection and its severe complications. Nevertheless, despite lack of solid prospective studies to support this hypothesis, several international guidelines were revisited, while postponing chemotherapy or elective surgery for specific indications in endemic areas were suggested [3]. A study performed in China indicated that among 1590 patients with confirmed COVID19, 18 were cancer patients, with lung cancer as the most frequent diagnosis [4]. Despite the very small sample size, the authors concluded the risk of COVID19 may be higher in cancer patients. Deasi et al. performed a meta-analysis using a random effects model to analyze the pooled prevalence of cancer among patients with COVID19 upon the current published observational cohorts. The authors found that the overall pooled prevalence of cancer in patients with COVID19 in these studies was 2.0% [5]. A prospective observational study from the United Kingdom evaluated 800 cancer patients with symptomatic COVID19. While more than half of the patients had mild COVID19 disease course, the risk for death was significantly associated with advanced patient age and comorbidities such as hypertension and cardiovascular disease [6]. Cancer patients who are on active anti-neoplastic treatment represent a heterogenous population, while various treatment modalities may differentially modify their immune milieu, including chemotherapy and immune checkpoint inhibitors (ICIs) [7]. Bersanelli and colleagues [8] recently suggested that ICI may restore cellular immunocompetence in cancer patients exposed to influenza infection. A second mechanism that may be induced by negative interference of ICI in the pathogenesis of COVID19 may involve alterations in the cytokine milieu. It has been shown that the T cell repertoire of symptomatic COVID19 patients demonstrated reduced counts of peripheral CD4 and CD8 T cells, although at a hyperactivate state [9]. Acute respiratory distress syndrome in COVID19 was characterized by abundant interstitial mononuclear inflammatory infiltrate in the lungs, dominated by lymphocytes, implying that immune hyperactivation mechanisms may play a key role in COVID19 severity [10]. In light of the altered immune milieu of cancer patients who are treated with immunomodulatory treatments, delineating the pattern of COVID19 infection in cancer patients is essential in order to tailor evidence-based recommendations for cancer care in the COVID19 era. We aimed to prospectively study a cohort of cancer patients in a tertiary cancer center over time compared with a cohort of oncology healthcare workers for COVID19 serology and clinical outcome. In infected individuals, we aimed to study immune profile to portray the pattern of immune response to COVID19.

## 2. Results

### 2.1. Participants

During the designated time period (29/3/2020–7/4/2020), 164 cancer patients on active intravenous treatment as well as 107 healthcare workers at the Rambam Health Care Campus (RHCC) oncology center were recruited into the study. Participant characteristics are delineated in Table 1 (additional classification of control cohort is described in Table S1). The patient group consisted of 72 males and 92 females, and the median age was 63 years. The control group consisted of 88 females and 19 males, and mean age was 41 years. Subjects in the control group were significantly more likely to reside with children, while those in the patient group were more likely to reside with older family members. Patients were exposed to fewer people during the study period, suggestive of self-isolation (*p* < 0.01). Staff were more likely to report COVID19-related symptoms in general when compared with patients, specifically cough (9.3% versus 1.2%, respectively, *p* < 0.01). Daily smoking was reported in 23% of patients and 12% of health workers, and co-morbidities were more frequent in the patient group, with 22% having hypertension and 17% with diabetes mellitus. To note, healthcare policy affirmed a universal facial masking as well as body temperature measuring in the hospital from mid-April 2020.

### 2.2. Disease- and Treatment-Related Characteristics

Of patients, 98 (60%) had metastatic disease and 40% had locoregional cancer. Lung metastases were documented in 22 patients (22%). Most common malignancies were breast (26%), lung (25%), and gastrointestinal (GI) cancer (25%). Treatments consisted of chemotherapy (74%), biological agents (33%), and immunotherapy (25%), while some patients received more than one treatment modality. Patient characteristics are described in Table 1.

### 2.3. COVID19 Clinical Status

During the study interval, there was no documented symptomatic case of COVID19 among the recruited participants, nor in the general patient population of the cancer center or in the healthcare workers cohort. Moreover, no COVID19-proven patients were reported out of the 8500 cancer patients who visited and/or were treated at the RHCC oncology center during the time of the pandemic outspread (between 12/2019 and 5/2020).

### 2.4. SARS-CoV2 Serological Status

**Figure 1:**
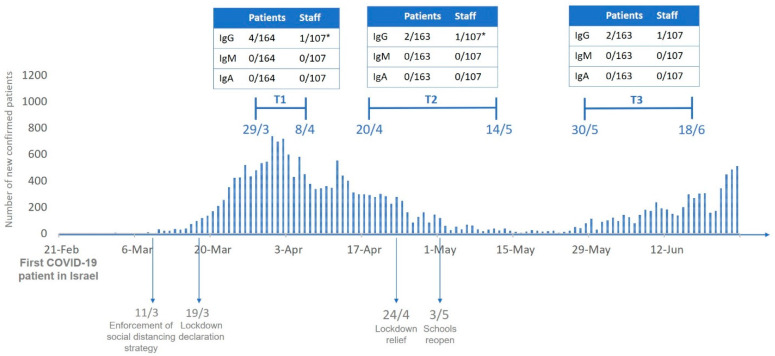
Scheme of the study timeline. * Different staff member at T1 and T2

**Figure 2:**
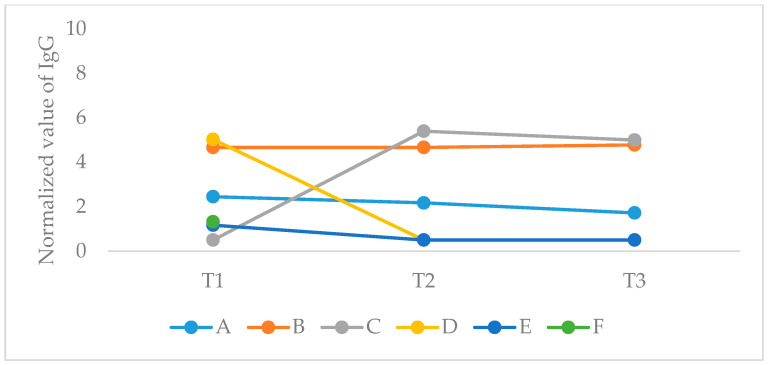
COVID19-positive serology values. Normalized values of SARS-CoV-2 IgG at the three examined time points. Positive expression was considered above the threshold value of 1.4. Participant F had only the T1 sample.

Overall, positive titer of anti-SARS-CoV-2 IgG was found at serum samples of 4 out of 164 patients and 2 out of 107 healthcare workers (one physician and one nurse) (Figure 1). Two of the patients were treated for local breast cancer, one for urinary cancer with lung metastases, and one for metastatic lung cancer. Out of the six participants with positive results, only one had COVID19-related co-morbidities (Table 2). IgG titer remained relatively stable throughout the study period in two participants. One positive case had been tested at T1 only due to technical issues. One healthcare worker had a negative titer at T1 and positive titer at T2 and T3, whereas two participants (one healthcare worker and one patient) had positive IgG titer at T1 and below threshold titer at T2 and T3 (Figure 2). Anti-SARS-CoV-2 IgM or IgA were undetectable in all samples at all time points (Figure 1).

### 2.5. CyTOF Analysis

**Figure 3:**
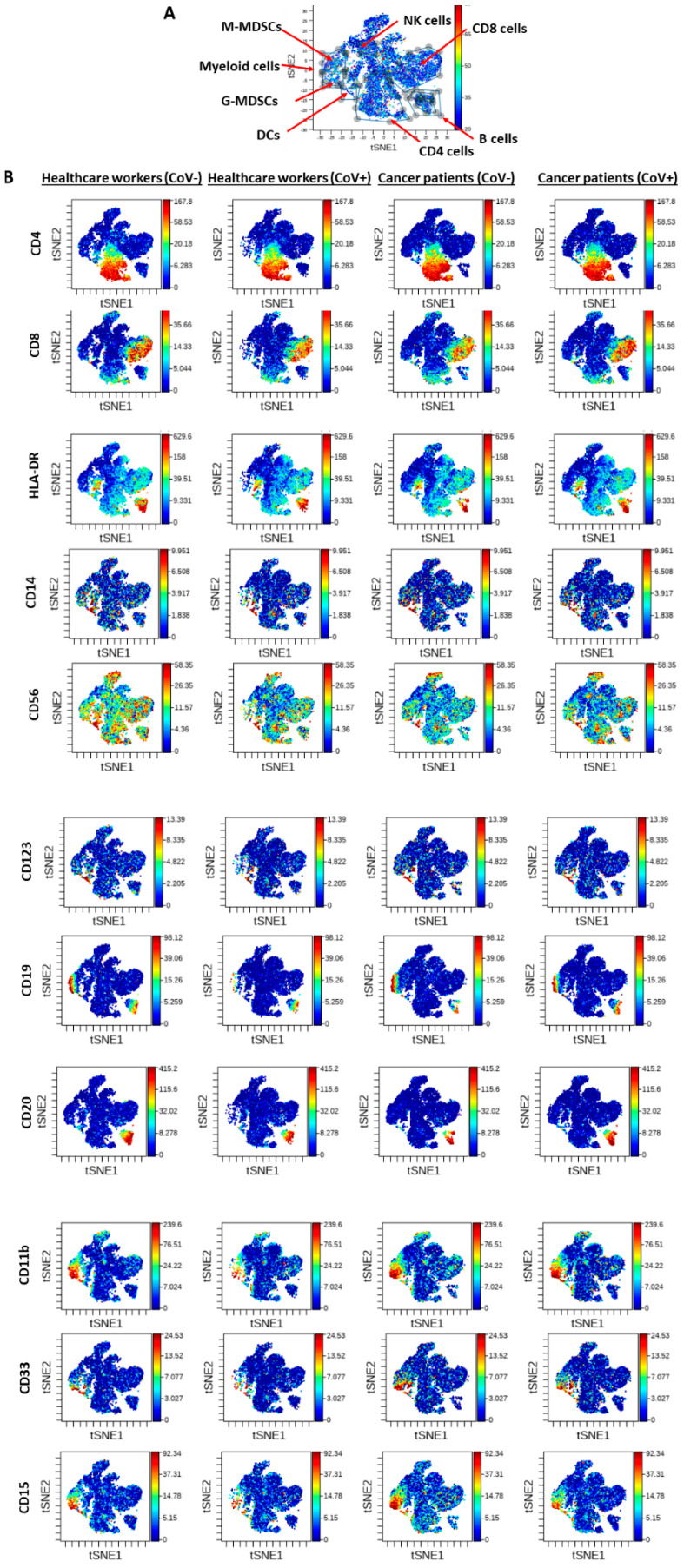
Immune composition of healthcare workers and cancer patients who are COVID19-positive or -negative. Peripheral blood samples from healthcare workers negative to SARS-CoV-2 IgG (CoV−, *n* = 6) or positive to SARS-CoV-2 IgG (*n* = 2) as well as cancer patients who were SARS-CoV-2 IgG-negative and -positive (*n* = 2 for each group, matched individuals) were acquired by mass cytometry (CyTOF). The samples were then grouped as indicated in the figure, and the percentages of various immune cells were analyzed as shown in Table 3. (**A**) A representative viSNE plot of the different immune cell population detected. (**B**) The specific surface markers representing the different immune cells, as indicated in Table S4, are shown for the four groups. DCs: dendritic cells, M-MDSCs: monocytic myeloid derived suppressor cells, G-MDSCs: granulocytic myeloid derived suppressor cells. In the viSNE plot, red represents high expression whereas blue represents low or no expression.

Analysis of the general population of immune cells yielded no significant changes in T cells between all four groups. Healthcare workers who were SARS-CoV-2 IgG+ exhibited a substantial reduction in CD8+ cells compared to all other groups. Furthermore, baseline levels of cancer patients who were SARS-CoV-2 IgG- displayed a major reduction in the composition of Natural killer (NK), dendritic, and B cells when compared to healthcare workers that were SARS-CoV-2 IgG-. Yet, interestingly, cancer patients and healthcare workers who were SARS-CoV-2 IgG+ displayed relatively the same levels of NK and B cells, but not dendritic cells (Figure 3, Table 3). Furthermore, when analyzing the myeloid lineage, we found a substantial increase in myeloid cells in cancer patients compared to healthcare workers (both SARS-CoV-2 IgG-), in line with previous studies [11,12]. However, a substantial reduction in myeloid cells, both monocytic and granulocytic cells, was observed in healthcare workers that were SARS-CoV-2 IgG+ compared to those who were SARS-CoV-2 IgG-, reaching 90% reduction in both cell populations, whereas in cancer patients the reduction in myeloid cells was relatively smaller, reaching approximately 50% reduction in those who were SARS-CoV-2 IgG+ compared to those who were SARS-CoV-2 IgG- (Figure 3, Table 3). These results further indicate the dramatic changes in cell composition between subjects who were positive and negative to the virus. These changes were observed mostly in the healthcare workers when compared to cancer patients. Interestingly, the changes in the percentage of immune cells between healthcare workers and cancer patients who were SARS-CoV-2 IgG- were also substantial when focusing on NK, dendritic, B, and myeloid cells, further suggesting that the immune cell composition may alter the sensitivity of cancer patients to the virus when compared to healthcare workers.

## 3. Discussion

Our study is the first to prospectively characterize longitudinal pattern of COVID19 infection among cancer patients on active anti-neoplastic treatment as well as healthcare providers at a tertiary cancer center. Once a national lockdown was announced in Israel on 15 March 2020, both cohorts experienced similar exposure levels, visiting only the cancer center, a secluded building within the main hospital campus (of 5300 employees). The study comprised a cohort of 164 consecutive patients who were treated during the last week of March 2020 who consented to the protocol and were followed until June 2020. The second cohort consisted of 107 healthcare providers who complied to enrolment into the study at the same time point. Our results indicate similar infection rate in both cohorts of about 2% asymptomatic cases in a longitudinal prospective study throughout a 2-month period with three points of serologic assessment. Out of 8500 cancer patients who had visited our cancer center between December 2019 to May 2020, no clinical symptomatic cases had been documented. Moreover, no symptomatic case had been reported among the 228 healthcare workers of the cancer center. It had been recently shown that there is a robust correlation between clinical severity of COVID19 infection and antibody titer since 2-week post-illness onset [13], which may entail a similar correlation in asymptomatic cases and rapid decline of antibody titer. Though it had been demonstrated that seroconversion following SARS-CoV-2 infection occurs usually 11–14 days after the first symptoms [14], there is no former evidence regarding dynamics in asymptomatic patients. To note, IgM and IgA levels that had been assessed in our study were undetectable at all three time points.

Several recent studies have addressed symptomatic COVID19 incidence among cancer patients. Dai et al. performed a multicenter study including 105 cancer patients and 536 age-matched non-cancer patients that were diagnosed with symptomatic COVID19 in China and indicated that cancer patients displayed more severe outcomes compared with non-cancer COVID19 patients. Risk factors for COVID19 severity were metastatic, hematological, and lung cancer. There was no difference between patients who were under active treatment or were not, and hence the authors concluded that cancer history may confer high risk to develop severe COVID19 disease [15]. In another study of 218 cancer patients with COVID19 in New York, increased mortality was significantly associated with older age, multiple comorbidities, need for intensive care unit support, and elevated levels of d-dimer and lactate dehydrogenase in multivariate analysis. Age-adjusted case fatality rates in patients with cancer compared with noncancer patients indicated a significant increase in case fatality for patients with cancer [16]. Analysis of comorbidities demonstrated increased risk of dying from COVID19 in patients with cancer with concomitant heart disease and chronic lung disease. Nevertheless, Lee et al. reported clinical outcomes of a larger cohort of 800 cancer patients with symptomatic COVID19 in the United Kingdom and indicated a lower mortality rate compared with the American study. Moreover, the only high-risk factors that were significantly correlated with COVID19 severity were patient age and cardiovascular comorbidities [6]. Therefore, current evidence is inconclusive and the potential role of cancer or anti-neoplastic treatments in COVID19 course remains to be elucidated. Evolving data regarding the immune profile in SARS-CoV2 infection shed light on a unique mechanism of action. Acute SARS-CoV2 infection results in broad changes in circulating immune cell populations—it induces local immune response within the lungs, recruiting macrophages and monocytes, cytokine release, and prime adaptive T and B cell immune responses. Due to increased secretion of the pro-inflammatory cytokine and chemokine T helper 1 (TH1), cell-polarized response is triggered and T lymphocytes, but not neutrophils, are attracted from the blood into the infected site [17]. A recent study described pathological findings in severe COVID19 and demonstrated the aberrant immune cell infiltrates found in the lung, resulting in lymphopenia and the increased neutrophil–lymphocyte ratio seen in the majority patients with SARS-CoV-2 infection [10]. It had been shown that the counts of peripheral CD4 and CD8 T cells were substantially reduced in COVID19-symptomatic patients, while their status was hyperactivated [17]. However, studies present heterogenous data regarding the immune response to SARS-CoV-2. While many COVID19 patients displayed robust CD8 T cell and/or CD4 T cell activation and proliferation, there was a subgroup of patients that had no detectable response compared to controls. In their recent study, Mathew et al. showcased that strong T and B cell activation and proliferation observed in a subset of COVID19 patients was durable and that the relative clinical lymphopenia was preferential for CD8 T cells with a lesser effect on CD4 T cells and almost no impact on B cells [18]. We have previously demonstrated in preclinical cancer models as well as in cancer patients that in response to anti-cancer therapy, the host mediates pro-tumorigenic and pro-metastatic activities [19,20]. These cellular host effects are accompanied by acute elevations of cytokines and growth factors generated by the host in response to the therapy. However, such effects are transient, and the host immediately reacts to the treatment by inducing counteractive activities by means of immunosuppression and anti-inflammatory cellular and molecular mechanisms, which in turn shift the immune states towards regeneration [19,20]. Overall, these phenomena indicate that cancer patients undergo immunological alterations towards anti-inflammation state due to anti-cancer treatments.

Due to the altered immune milieu, we sought to evaluate COVID19 dynamics throughout the pandemic among cancer patients. We performed immune profiling of the SARS-CoV-2 IgG+ subjects in our study compared with age-matched, co-morbidity matched SARS-CoV-2 IgG- subjects from both cohorts. For the cancer cohort, SARS-CoV-2 IgG- cases for immune profiling were selected upon the treatments of the positive cases. Limited by a small sample size of positive cases, our results imply a differential immune profile for the cancer patients compared with healthy subjects.

SARS-CoV-2 IgG+ non-cancer subjects exhibited major changes when compared with cancer patients, who displayed relatively modest changes, when we compared patients who were positive or negative to COVID19 infection. Interestingly, in the myeloid lineage, the reduction in myeloid cells in the healthcare workers was substantially reduced (over 90% reduction in SARS-CoV-2 IgG+ group), whereas in SARS-CoV-2 IgG+ cancer patients, reduction was moderate (approximately 50%) compared with matched SARS-CoV-2 IgG-. As myeloid cells and especially myeloid-derived suppressor cells (MDSCs) are key players in reducing cytotoxic immune cell activation [21], this may imply reduction of cytokine secretion in cancer patients when compared to the overall population.

Limited by the very small sample size, our preliminary results indicate that cancer may exhibit differential host response to SARS-CoV2 infection. Along with the very low incidence of COVID19 in our cohort in a longitudinal serologic study, as well as no documentation of any clinical symptomatic COVID19 case in a larger cohort of 8500 patients who were actively observed in the last 6 months in our center, our study indicates that the immunological perspective of cancer treatments on the risk for COVID19 infection should be further explored. Another limitation of the study is the lack of symptomatic COVID19 cases (of hospitalized non-cancer patients) as positive controls, as such individuals were not amenable to be enrolled into the study.

Delineating prospective COVID19 infection trends and patterns in a larger cohort of cancer patients who are on active anti-cancer treatment will lend credence to tailor future healthcare policy in the current unpredictable COVID19 era.

## 4. Materials and Methods

### 4.1. Participants and Design

The study included cancer patients receiving intravenous treatment administered at the infusional ambulatory unit of the oncology center or inpatient service (for continuous chemotherapy protocols), as well as healthcare workers from the same oncology center within the Rambam Health Care Campus, Haifa, Israel. During the study period (3/2020–6/2020), blood was drawn at three time points and separated for peripheral blood mononuclear cells (*PBMCs*) and serum. Participants were asked to complete questionnaires for information on their sociodemographic status, potential exposure to COVID19, and related symptoms at the same time points. The study protocol was approved by the Institutional Ethics Committee of Rambam Health Care Campus (RHCC; RMB 0209-20). Clinical data for cancer patients’ characteristics was retrieved from the RHCC electronic medical records.

### 4.2. COVID19 Serology

Serum samples were analyzed at three time points for the detection of anti-COVID19 antibodies. For IgG expression, we used anti-SARS-CoV-2 IgG assay (Abbott, Abbott Park, IL, USA), a chemiluminescent microparticle qualitative immunoassay (CMIA) detected in ARCHITECT *i* system with a cutoff result of 1.4 (positive result > 1.4). The anti-SARS-CoV-2 IgG assay gained Food and Drug Administration (FDA) Emergency Use Authorization (EUA). IgM expression was detected using an EDI Novel Coronavirus COVID19 IgM ELISA qualitative detection assay (Epitope Diagnostics Inc., San Diego, CA, USA), and IgA expression was detected using anti-SARS-CoV-2 ELISA (IgA) semi-quantitative assay (EUROIMMUN AG, Luebeck, Germany). All serological tests were conducted at Rambam virology diagnostic unit.

### 4.3. Time of Flight Mass Cytometry (CyTOF)

High throughput mass cytometry (CyTOF) analysis of immune cell composition was performed as previously described [19]. Briefly, peripheral blood cells from participants, at the second time point of the study were selected for CyTOF analysis as follows: staff who were SARS-CoV-2 IgG- (*n* = 6); staff who were SARS-CoV-2 IgG+ (*n* = 2; cases were matched upon gender and age). Cancer patients who were SARS-CoV-2 IgG+ (*n* = 4) were matched to cancer patients who were SARS-CoV-2 IgG- in terms of age, gender, cancer type, and treatment protocol (*n* = 4). Samples were excluded from the CyTOF analysis if technical errors occurred at the time of acquisition or when the number of PBMC collected was very low to appropriately analyze the samples (*n* = 2; cancer patients, characterization of patients’ disease status, and characterization of treatment is described at Table S2). All samples from the four groups were separately acquired by CyTOF, and subsequently were grouped for the analysis. All peripheral blood samples underwent red blood cell lysis. Subsequently, cells were counted and immunostained with a mixture of metal-tagged antibodies using the different surface markers, as indicated in Table S3. All antibodies were conjugated using the MAXPAR reagent (Fluidigm, San Francisco, CA, USA) and tittered prior to staining. Cells were washed twice with PBS, fixed in 1.6% formaldehyde (SigmaAldrich, St. Louis, MO, USA), washed again in ultrapure H_2_O, and acquired by CyTOF mass cytometry system (Fluidigm, San Francisco, CA, USA). Acquired data was uploaded to Cytobank web server (Cytobank Inc., Santa Clara, CA, USA). CD45+ cells were used to gate on immune cells, and the gated cells were segregated into sub-population clusters by expression markers. The specific immune cell types were defined on the basis of the expression of surface markers shown in Table S4. Data analysis was performed by viSNE algorithm [20], via the Cytobank online platform at https://www.cytobank.org/.

### 4.4. Statistical Analysis

Statistical analyses were performed using SPSS statistical software. All tests were two-sided with a significance level of α = 0.05. Chi square and Mann–Whitney tests were used for demographic data analysis.

## 5. Conclusions

Despite lack of concrete evidence, cancer patients were considered at the onset of the COVID19 pandemic as a high-risk population for COVID19 infection. However, current evidence is inconclusive and the potential role of cancer or anti-neoplastic treatments in COVID19 course remains to be elucidated.

Our results may indicate that due to differential immune cell profile of cancer patients who are treated with immunomodulatory agents, the host response to the SARS-COV2 may lessen symptom severity. Delineating COVID19 infection trends in asymptomatic healthcare workers as well as a cohort of cancer patients who are on active anti-cancer treatment will lend credence to tailor future healthcare policy in the next phases of the pandemic.

## Figures and Tables

**Table 1:** Participant-, disease-, and treatment-related characteristics.

Characteristics	Patients	Staff
**Gender (No.)**		
Male	72	19
Female	92	88
**Age (Years)**		
Median	63	41
Range	(23–90)	(20–73)
**Isolation**		
Due to foreign travel	1%	2%
Due to COVID19 exposure	1%	1%
**Living arrangement**		
No. people at home—median	1	3
No. of people at home—range	(0–2)	(0–9)
Lives with people above age 70	26%	3%
Lives with people under age 18	23%	56%
**Daily exposure to no. of people**		
Median	0	30
Range	(0–10)	(2–100)
Report of COVID19 symptoms	7%	9%
**Co-Morbidities**		
Hypertension	22%	9%
Diabetes mellitus	17%	0%
Hyperlipidemia	9%	1%
Heart disease	8%	1%
Lung disease	1%	0%
Asthma	2%	6%
**Smoking**		
Daily smoker	23%	12%
Former smoker (<5 years ago)	12%	11%
Former smoker (>5 years ago)	24%	11%
Never smoker	40%	60%
**Cancer type**		
Breast	26%	NR
Lung	25%	NR
Gastrointestinal	25%	NR
Genitourinary	7%	NR
Nervous system	5%	NR
Head and neck	4%	NR
Melanoma	4%	NR
Gynecological	2%	NR
Sarcoma	2%	NR
**Disease stage**		
Metastatic disease	60%	NR
Local disease	40%	NR
Lung metastases	22%	NR
**Type of treatment**		
Chemotherapy	74%	NR
Biological therapy	33%	NR
Immunotherapy	25%	NR

No.—number; NR—not relevant.

**Table 2:** Description of COVID19 serology-positive patients.

Patient	Gender	Age	Type of Cancer	Stage	Lung Metastasis	Treatment	HTN	DM	Asthma	Smoking	VTE
1	Female	65	Breast	Local		Paclitaxel					None
2	Female	53	Breast	Local		Tratsuzumab					None
3	Male	69	Urinary	Metastatic	V	Pembrolizumab					None
4	Female	70	Lung	Metastatic		Carboplatin + vp-16	V	V	V	Former smoker (<5)	None

HTN—hypertension; DM—diabetes mellitus VTE—venous thromboembolism; (<5)—less than 5 years.

**Table 3:** The relative changes in the percentage of immune cells in different human subjects positive or negative to SARS-CoV-2 IgG. CyTOF analysis was performed on peripheral blood cells from staff and cancer patients either negative to SARS-CoV-2 IgG (CoV−) or positive to it (CoV+). The percentages in the table are after samples were grouped.

Human Subject	CD4 Cells	CD8 Cells	NK Cells	DC Cells	B Cells	Myeloid Cells	M-MDSCs	G-MDSCs
**Staff (CoV−)**	23.39%	24.76%	4.88%	2.08%	7.26%	10.76%	8.80%	1.85%
**Staff (CoV+)**	25.26%	13.02%	2.22%	0.84%	5.72%	0.77%	0.44%	0.26%
**Cancer patients (CoV−)**	20.27%	21.66%	1.35%	0.42%	0.82%	17.88%	9.05%	8.08%
**Cancer patients (CoV+)**	23.25%	22.24%	2.25%	0.49%	6.46%	7.79%	2.93%	4.68%

## Supplementary Materials

The following are available online at https://www.mdpi.com/2072-6694/12/9/2421/s1, Table S1: Classification of healthcare-workers’ cohort. Table S2: Characteristics of CyTOF cancer patients’ samples. Table S3: The antibody panel used for CyTOF. Table S4: The definition of immune cells based on surface markers.
