# Supplementary material for: The Potential Role of Immune Alteration in the Cancer–COVID19 Equation—A Prospective Longitudinal Study
Source: Cancers (Basel). 2020 Aug 26;12(9):2421. doi: 10.3390/cancers12092421 (PMC7563644; doi:10.3390/cancers12092421)
Supplement: Supplementary file 1 [file cancers-12-02421-s001.pdf]

**Table S1: Classification of healthcare-workers' cohort**

| | Total | Inpatient unit | Outpatient unit | Both units |
|---|---|---|---|---|
| **Gender** | | | | |
| Female | 89 | 19 | 68 | 2 |
| Male | 18 | 11 | 4 | 3 |
| **Age** | | | | |
| Median | 41 | 41 | 41 | 39 |
| Range | (20-73) | (24-73) | (20-64) | (31-60) |
| **Professions** | | | | |
| Physicians | 10 | 4 | 3 | 3 |
| Nurses | 53 | 25 | 28 | |
| Secretaries | 14 | 1 | 13 | |
| Research coordinators | 21 | | 21 | |
| Social workers | 4 | | 4 | |
| Medical Assistants | 1 | | 1 | |
| Directors | 4 | | 2 | 2 |

**Table S2: Characteristics of CyTOF cancer patients' samples**

| Patient | Gender | Age | Type of Cancer | Stage | Treatment | SARS-CoV-2 Ab +/- |
|---|---|---|---|---|---|---|
| 1 | Female | 65 | Breast | Local | Paclitaxel | Positive |
| 2 | Female | 69 | Breast | Local | Paclitaxel | Negative |
| 3 | Male | 69 | Urinary | Metastatic | Pembrolizumab | Positive |
| 4 | Female | 48 | Lung | Metastatic | Pembrolizumab | Negative |

Description of disease status and treatment of the cancer patients SARS-CoV-2 IgG negative and positive samples who were analysed by CyTOF.

**Table S3: The antibody panel used for CyTOF**

| # | Metal | Ab1 | Cat# | Clone | Supplier | Supp. cat |
|---|---|---|---|---|---|---|
| 1 | 112Cd | CD45 BCF | 029 | HI30 | Biolegend | 304002 |
| 2 | 113In | CD44 BCF | 007 | IM7 | Biolegend | 103014 |
| 3 | 115In | CD8a | 517 | RPA-T8 | Biolegend | 301053 |
| 4 | 116Cd | CD19 | 799 | HIB19 | Biolegend | 302247 |
| 5 | 141Pr | CD57 | 775 | HNK-1 | Biolegend | 359602 |
| 6 | 142Nd | CD11c | 776 | 3.9 | Biolegend | 301602 |
| 7 | 143Nd | CD117 | 583 | 104D2 | Fluidigm | 3143001B |
| 8 | 144Nd | CD34 | 759 | 581 | Biolegend | 343531 |
| 9 | 145Nd | CD4 | 778 | RPA-T4 | Biolegend | 300541 |
| 10 | 146Nd | CCR5 | 7 | CTC5 | R&D Systems | MAB1802 |

| # | Metal | Ab1 | Cat# | Clone | Supplier | Supp. cat |
|---|---|---|---|---|---|---|
| 11 | 147Sm | CD20 | 699 | 2H7 | Biolegend | 302343 |
| 12 | 148Nd | CD62L | 742 | DREG-56 | Biolegend | 304835 |
| 13 | 149Sm | CD7 | 535 | CD7-6B7 | Biolegend | 343102 |
| 14 | 150Nd | IgD | 791 | IA6-2 | Biolegend | 348235 |
| 15 | 151Eu | CD209 | 68 | DCS-8C1 | Biolegend | 343002 |
| 16 | 152Sm | CD27 | 162 | O323 | Biolegend | 302802 |
| 17 | 153Eu | CD45RA | 585 | HI100 | Fluidigm | 3153001B |
| 18 | 154Sm | CD69 | 63 | FN50 | Biolegend | 310902 |
| 19 | 155Gd | CD25 | 72 | BC96 | Biolegend | 302602 |
| 20 | 156Gd | CD10 | 428 | HI10a | Fluidigm | 3156001B |
| 21 | 157Gd | PDL-1 | 358 | 29E.2A3 | Biolegend | 329719 |
| 22 | 158Gd | CD123 | 751 | 6H6 | Biolegend | 306027 |
| 23 | 159Tb | CD33 | 783 | WM53 | Biolegend | 303419 |
| 24 | 160Gd | CD14 | 719 | M5E2 | Biolegend | 301843 |
| 25 | 161Dy | CXCR5 | 815 | J252D4 | Biolegend | 356902 |
| 26 | 162Dy | CD11b | 159 | ICRF44 | Biolegend | 301312 |
| 27 | 163Dy | CD127 | 566 | A019D5 | Biolegend | 351302 |
| 28 | 164Dy | CD15 | 435 | W6D3 | Fluidigm | 3164001B |
| 29 | 165Ho | CD45RO BCF | 015 | UCHL1 | Biolegend | 304202 |
| 30 | 166Er | CD16 | 86 | 3G8 | Biolegend | 302014 |
| 31 | 167Er | CD38 | 765 | HIT2 | Biolegend | 303535 |
| 32 | 168Er | CCR6 (weak) | 725 | G034E3 | Biolegend | 353427 |
| 33 | 169Tm | CCR7 (?) | 90 | G043H7 | Biolegend | 353202 |
| 34 | 170Er | CCR4 | 93 | 2G12 | Biolegend | 131202 |
| 35 | 171Yb | CD66b | 689 | G10F5 | Biolegend | 305102 |
| 36 | 173Yb | CD3 | 762 | UCHT1 | Biolegend | 300443 |
| 37 | 174Yb | HLA-DR | 581 | L243 | Fluidigm | 3174001B |
| 38 | 175Lu | PD-1 | 801 | H12.2H7 | Fluidigm | 3175008B |
| 39 | 176Yb | CD56 | 582 | CMSSB | Fluidigm | 3176003B |

A list of the full panel of metal-conjugated antibodies used for CyTOF. Antibodies were in a carrier-free format. Conjugation was performed using the MAXPAR Conjugation Kit.

**Table S4: The definition of immune cells based on surface markers**

| Cell Type | Surface markers* |
|---|---|
| T helper cells | CD4+ |
| Cytotoxic T cells | CD8+ |
| NK cells | HLA-DR- CD14- CD56+ CD123- |
| Dendritic cells | HLA-DR+ CD14- |
| B cells | CD19+ CD20+ |
| Myeloid cells | HLA-DR+ CD11b+ CD33+ |
| Monocytic myeloid cells | HLA-DR- CD11b+ CD14+ |
| Granulocytic myeloid cells | HLA-DR- CD11b+ CD14- CD15+ |

*The definition of cells includes the pan-hematopoietic marker CD45.
